# Treatment of Short Stature in Aggrecan-deficient Patients With Recombinant Human GH: 3-year Response

**DOI:** 10.1210/jendso/bvae177

**Published:** 2024-10-10

**Authors:** Gajanthan Muthuvel, Andrew Dauber, Eirene Alexandrou, Leah Tyzinski, Vivian Hwa, Philippe Backeljauw

**Affiliations:** Division of Endocrinology, Cincinnati Children's Hospital Medical Center, Cincinnati, OH 45229, USA; Department of Pediatrics, University of Cincinnati, Cincinnati, OH 45267, USA; Division of Endocrinology, Children's National Hospital, Washington, DC 20010, USA; Department of Pediatrics, George Washington University School of Medicine and Health Sciences, Washington, DC 20052, USA; Division of Endocrinology, The University of Iowa Stead Family Children's Hospital, Iowa City, IA 52242, USA; Department of Pediatrics, University of Iowa, Iowa City, IA 52242, USA; Division of Endocrinology, Cincinnati Children's Hospital Medical Center, Cincinnati, OH 45229, USA; Division of Endocrinology, Cincinnati Children's Hospital Medical Center, Cincinnati, OH 45229, USA; Department of Pediatrics, University of Cincinnati, Cincinnati, OH 45267, USA; Division of Endocrinology, Cincinnati Children's Hospital Medical Center, Cincinnati, OH 45229, USA; Department of Pediatrics, University of Cincinnati, Cincinnati, OH 45267, USA

**Keywords:** Aggrecan deficiency, short stature, growth hormone

## Abstract

**Context:**

Patients with aggrecan (ACAN) deficiency present with dominantly inherited short stature, as well as early-onset joint disease.

**Objective:**

The objective of this study was to evaluate the efficacy and safety of recombinant human GH (rhGH) on linear growth in ACAN-deficient children.

**Methods:**

Open-label, single-arm, prospective study over 3 years recruiting 10 treatment-naïve patients with heterozygous mutations in *ACAN*, age ≥2 years, prepubertal, and normal IGF-I concentration. Patients were treated with rhGH (initially, 50 mcg/kg/day). Main outcomes were change in (Δ) height SD score (HtSDS) and height velocity (HV).

**Results:**

Ten patients (6 females) enrolled with median chronological age (CA) of 5.6 years (range, 2.4-9.7). Baseline median HtSDS, HV, and bone age/CA were −2.5 (range, −4.3 to −1.1), 5.2 cm/year (range, 3.8 to 7.1), and 1.2 (range, 0.9 to 1.5), respectively. The cumulative median ΔHtSDS over 3 years was +1.21 (range, +0.82 to +1.94). Median HV increased to 8.3 cm/year (range, 7.3-11.2), 7.7 cm/year (range, 5.9-8.8), and 6.8 cm/year (range, 4.9-8.6) during years 1, 2, and 3, respectively. The median Δ predicated adult height was +6.8 cm over 3 years. Four female subjects entered puberty; nevertheless, median Δbone age/CA was −0.1. No adverse events related to rhGH were observed.

**Conclusion:**

Linear growth improved in a cohort of ACAN-deficient patients treated with rhGH, albeit somewhat attenuated in older participants who entered puberty. Longitudinal follow-up is needed to assess the long-term efficacy of rhGH and adult height outcome.

Aggrecan (ACAN) is a proteoglycan located in the extracellular matrix of articular and growth plate cartilage encoded by the *ACAN* gene [1-4]. Animal studies have shown variants in *ACAN* result in altered growth plate morphogenesis, contributing to premature hypertrophic chondrocyte maturation and impaired long bone elongation [2, 4]. Patients with heterozygous pathogenic variants in *ACAN* have classically been described to present with short stature, advanced skeletal maturation, and premature epiphyseal fusion leading to early growth cessation [5-8]. It has more recently been shown that some individuals with ACAN deficiency may present without bone age (BA) advancement [9-11]. Early-onset osteoarthritis has commonly been observed in adults [6, 9], whereas children may present with subtle disproportionality and osteochondritis dissecans (OD) [9, 12].

The evaluation of short stature has evolved to incorporate molecular genetic testing, resulting in reclassification of patients previously diagnosed with idiopathic short stature with specific genetic etiologies [5, 13-15], including ACAN deficiency. Although ACAN deficiency has become increasingly recognized [5-11, 16-18], information on the effects of growth-promoting interventions have largely been limited to retrospective reviews [6, 7, 10, 16, 19] and/or patient cohorts that may have been treated with different GH regimens of varying duration, with or without puberty blockade [6, 7, 10, 16, 18-21], thus making it difficult to determine the efficacy of the treatment.

The objective of this study was to perform a prospective trial of recombinant human GH (rhGH) therapy in ACAN-deficient patients to evaluate its efficacy and safety. We previously reported results of the first year of treatment in this trial [12], subsequently extended for another 2 years and reported here.

## Patients and Methods

The institutional review board at Cincinnati Children's Hospital Medical Center (Cincinnati, OH) approved the trial. Written informed consent was obtained from parents of all subjects and assent from subjects of relevant age. Ten treatment-naïve prepubertal patients with genetically confirmed ACAN deficiency were treated with rhGH for 3 years in an open-label, single-arm prospective study.

### Study Design

A detailed study protocol was documented in the prior 1-year report [12] and extended to a 3-year trial. Inclusion criteria were: heterozygous pathogenic variant in *ACAN* (defined as either a heterozygous deletion of the entire gene or of at least 1 complete exon, any truncating variant, any missense variant predicted to be damaging and *de novo* or segregating with the short stature familial phenotype, and in-frame insertions or deletions of more than one amino acid and meeting same criteria as missense mutations), age ≥ 2 years, prepubertal examination, and an IGF-I concentration within normal range for age and gender. Exclusion criteria were: previous growth-altering treatment (including rhGH, rhIGF-1, GnRH agonist, aromatase inhibitor, oxandrolone, or any investigational pharmacologic intervention targeting growth), history of malignancy, other chronic conditions known to impact growth (eg, cystic fibrosis, inflammatory bowel disease, celiac disease, asthma requiring significant inhaled steroid use), malnutrition (body mass index [BMI] <5th percentile), and fusion of growth plates as determined on BA.

Study visits were conducted every 6 months, with collection of auxologic data and physical examination. Height and weight data were plotted on Centers for Disease Control and Prevention growth charts (www.cdc.gov/growthcharts). Sitting height to standing height SD scores (SDS) were calculated from published reference data derived from the National Health and Nutrition Examination Survey III by Hawkes et al [22]. Height velocity (HV) SDS were obtained from published references, Kelly et al [23], and for younger participants less than 6 years old, Prader et al [24]. Baseline complete blood count, renal and hepatic panel, thyroid function studies, IGF-I, and IGF binding protein-3 (IGFBP-3) were confirmed normal upon enrollment. IGF-I and IGFBP-3 were measured using the Immunodiagnostic Systems (Immunodiagnostics System Inc, Gaithersburg, MD) IDS-iSYS chemiluminescent immunoassay kits (Cat# IS-3900, RRID:AB_2861357 and Cat# IS-4400, RRID:AB_2895663, respectively). IGF-I SDS was calculated using the formula [(IGF-I/µ)^λ^] – 1//(λ×σ), with variables provided for age and sex as in Bidlingmaier et al [25]. For IGFBP-3 a modified LMS method (skewness [L], mean [M], and coefficient of variation [S]) was also used to calculate SDS [26].

Subjects were initiated on rhGH at a dose of 50 mcg/kg/day subcutaneously, modeled off dosing for other primary skeletal disorders [27]. The rhGH was provided by Novo Nordisk, Inc. (Norditropin and Nordiflex). IGF-I and IGFBP-3 were monitored while on rhGH every 6 months and adherence with rhGH administration was monitored via compliance log reviews and tracking of GH pen usage. Dose reductions in rhGH of 10% to 20% were made for IGF-I concentrations > 2.5 SDS above the mean. Dual x-ray absorptiometry (Hologic Inc., Bedford, MA) of the lumbar spine and total body, as well as BA x-rays (determined by the Greulich and Pyle method), were obtained at baseline and annually. Predicted adult height (PAH) was estimated utilizing BoneXpert (Visiana, Holte, Denmark; www.boneXpert.com). Magnetic resonance imaging of the knees for patients older than age 6 years who did not require sedation, as well as x-rays of the knees, were obtained as part of a separate phenotyping protocol, published elsewhere [9]

### Outcomes

The primary treatment outcomes were change in (Δ) height SD score (HtSDS) over the 3-year study duration and ΔHV. Secondary outcomes included change in BA in comparison to chronologic age (CA). Statistical analysis was completed using SAS, version 9.4 (SAS Institute, Cary, NC), with results reported for both paired *t*-tests as well as nonparametric signed-rank test, using the latter to determine statistical significance. A statistically significant difference was indicated by a *P* ≤ .05. In addition, the frequency of adverse events was determined.

## Results

Ten subjects with a confirmed heterozygous pathogenic variant in *ACAN* were enrolled with a median CA of 5.6 years (range, 2.4-9.7 years) (Table 1). Median baseline BA was 6.9 years (range, 2.5-10.0 years), with median BA/CA of 1.2 (range, 0.9-1.5). Baseline median HtSDS was −2.52 (range, −4.27 to −1.07), median weight SDS was −0.67 (range, −3.09 to +1.47), and BMI percentile was 86.2 (range, 36.2-98.8). Sitting height/standing height (SH/Ht) measurements at baseline showed a spectrum of disproportionality, with 4 subjects showing increased SH/Ht assessments (SDS > 2) and median SH/Ht SDS of +1.80 (range, −1.4 to +4.2).

**Table 1.** Aggrecan variants and baseline chronological age and bone age for 10 enrolled study patients

Patient/gender	cDNA (protein)	CA (year)	BA (year)
P1/F	c.1172delG (p.Gly391Valfs*7)	2.4	2.5
P2/M	c.280_336del57 (p.Val94_Ile112del)	3.4	5.0
P3/M	c.2023C>T (p.Arg675*)	3.6	5.0
P4/M	c.280_336del57 (p.Val94_Ile112del)	5.0	6.0
P5/F	c.7202G>A (p.Trp2401*)	5.2	6.8
P6/M	c.609G>T (p.Trp203Cys)	6.0	7.0
P7/F	c.7202G>A (p.Trp2401*)	6.9	7.8
P8/F	c.492C>A (p.Tyr164*)	7.4	9.4
P9/F	c.1051 + 1G>A (p.Val255_Glu352del)	8.1	10.0
P10/F	c.2023C>T (p.Arg675*)	9.7	8.8
Mean (SD)	—	5.8 (2.3)	6.8 (2.3)
Median [range]	—	5.6 [2.4, 9.7]	6.8 [2.5, 10.0]

Abbreviations: BA, bone age; CA, chronological age; F, female; M, male.

### Response to rhGH Therapy

**Figure 1.**
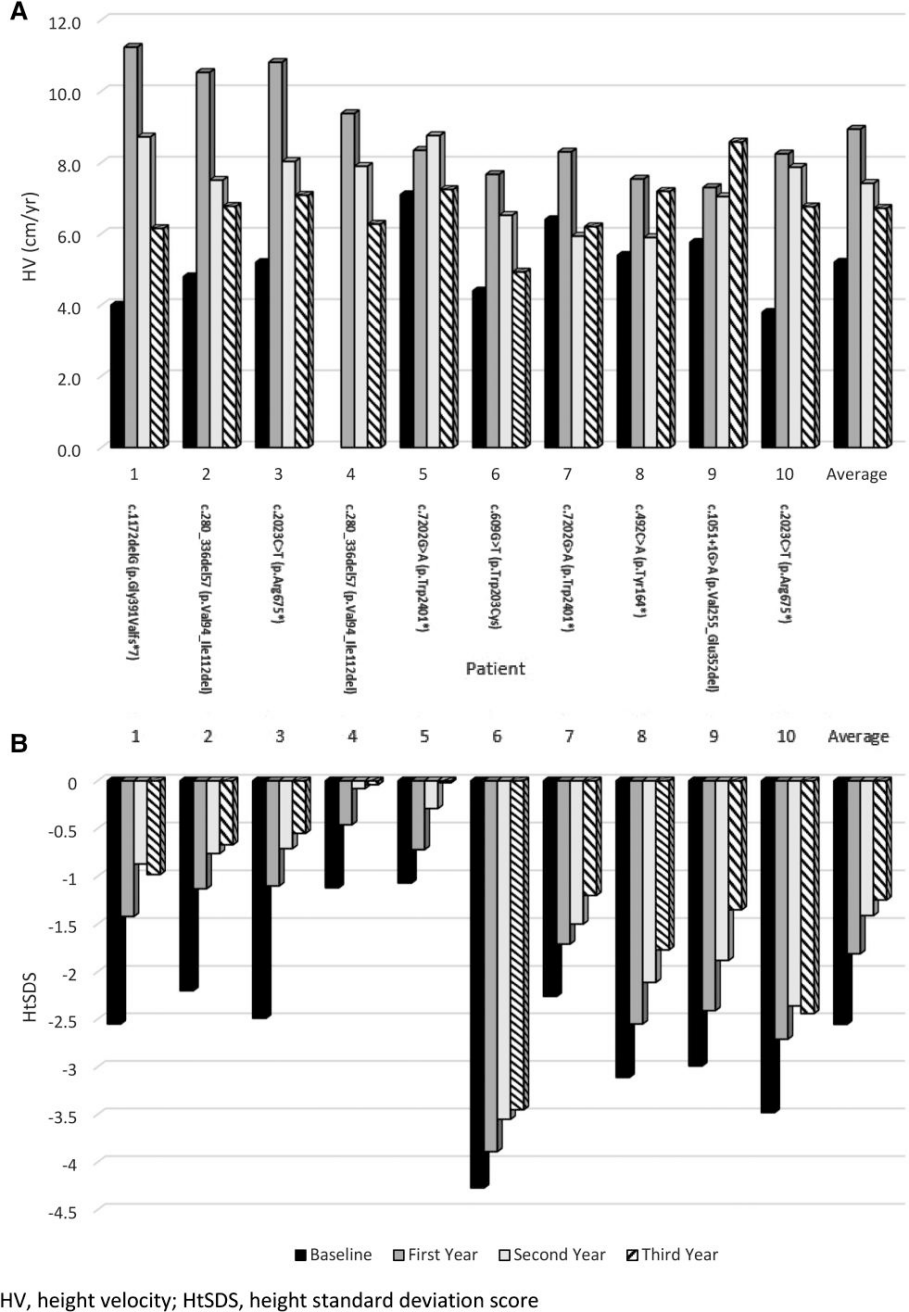
Linear growth response in 10 patients with aggrecan (ACAN) deficiency treated with recombinant human GH, displayed in order of chronological age with *ACAN* variants included. (A) Pretreatment height velocity (black bars) and height velocity through 1 year (dark gray bars), 2 years (light gray bars), and 3 years (striped bars) of treatment with recombinant human GH (rhGH). Median height velocity increased from 5.2 cm/year (range, 3.8-7.1 cm/year) at baseline to 8.3 cm/year (range, 7.3-11.2 cm/year) during the first year of treatment, 7.7 cm/year (range, 5.9-8.8 cm/year) during the second year of treatment, and 6.8 cm/year (range, 4.9-8.6 cm/year) during the third year of treatment (*P* = .012). (B) Height SD score (SDS) in 10 subjects, before (black bars) and after 1 year (dark gray bars), 2 years (light gray bars), and 3 years (striped bars) of treatment with rhGH. Median change in (Δ) HtSDS after 1 year was +0.62 (range, +0.35 to +1.39), +0.39 (range, +0.21 to +0.55) in the second year, and +0.13 (range, −0.11 to +0.53). The cumulative median ΔHtSDS was +1.21 (range, +0.82 to +1.94) (*P* = .002).

HV and HtSDS at baseline and through 3 years of treatment with rhGH are shown in Fig. 1. The median HtSDS increased from −2.52 at baseline (range, −4.27 to −1.07) to −1.57 (range, −3.89 to −0.46) after 1 year of treatment, −1.19 (range, −3.55 to −0.08) after 2 years of treatment, and −1.09 (range, −3.45 to −0.02) after 3 years of treatment. As demonstrated in Table 2, the median ΔHtSDS from baseline through 3 years of treatment was +1.21 (range, +0.82 to +1.94) (*P* = .002), with ΔHtSDS of +0.62 (range, +0.35 to +1.39) during the first year, +0.39 (range, +0.21 to +0.55) in the second year, and +0.13 (range, −0.11 to +0.53) during the third year. Median pretreatment HV was 5.2 cm/year (range, 3.8-7.1 cm/year). This increased to 8.3 cm/year (range, 7.3-11.2 cm/year) during the first year of treatment, 7.7 cm/year (range, 5.9-8.8 cm/year) during the second year of treatment, and 6.8 cm/year (range, 4.9-8.6 cm/year) during the third year of treatment. PAH increased over the 3 years, with median ΔPAH of +6.8 cm (range, +3.8 to +9.1) (*P* = .002). There was no consistent change in proportionality among the cohort from baseline to end of treatment course, with median ΔSH/Ht SDS of −0.06 (range, −2.67 to +1.92) (*P* = .770). As seen in Table 2, there was no statistically significant change in BMI, given median ΔBMI percentile of +0.77 (range, −20.20 to +28.88) (*P* = .695).

**Table 2.** Change in height SD score (HtSDS), height velocity (HV), bone age, predicted adult height (PAH), sitting height, body mass index (BMI), and bone density measures after 3 years of treatment with recombinant human GH

	Δ HtSDS	Δ Height velocity (cm/year)	Δ Height velocity SDS	Δ Bone Age/chronological age*^a^* (year)	Δ Predicted adult height (cm)*^a^*	Δ SH/Ht SDS*^a^*	Δ BMI Percentile	Δ LS BMD HAZ*^a^*	Δ TBLH BMD HAZ*^a^*
P1	+1.57	+2.1	+4.5	−0.02 (1.04, 1.02)	+7.4 (150.1, 157.5)	+0.75 (+0.76, +1.51)	−0.53 (88.71, 88.18)	N/A (N/A, +1.5)	N/A (N/A, +0.8)
P2	+1.53	+2.0	+2.9	−0.27 (1.47, 1.20)	+5.9 (161.8, 167.7)	−2.25 (+0.01, −2.24)	−20.20 (97.49, 77.29)	N/A (N/A, +0.3)	N/A (N/A, −0.3)
P3	+1.94	+1.9	+3.0	−0.08 (1.39, 1.31)	+8.8 (158.7, 167.5)	−1.77 (+2.99, +1.22)	−2.54 (96.36, 93.82)	N/A (N/A, +1.0)	N/A (N/A, +0.6)
P4	+1.08	N/A	N/A	+0.18 (1.20, 1.38)	+3.8 (166.1, 169.9)	+1.68 (+1.82, +3.50)	+1.20 (98.79, 99.99)	−0.8 (+2.2, +1.4)	−0.1 (+0.5, +0.4)
P5	+1.05	+0.1	+0.6	−0.25 (1.31, 1.06)	+9.1 (152.8, 161.9)	+1.92 (−1.42, +0.50)	+1.42 (72.47, 73.89)	+0.1 (−0.3, −0.2)	−0.2 (−0.8, −1.0)
P6	+0.82	+0.5	+1.9	+0.03 (1.17, 1.20)	+4.7 (145.0, 149.7)	−0.18 (+2.46, +2.28)	+2.53 (79.09, 81.62)	+1.0 (−0.5, +0.5)	−0.4 (0.0, −0.4)
P7	+1.06	−0.2	−0.3	−0.12 (1.13, 1.01)	+7.2 (147.4, 154.6)	+0.06 (+1.56, +1.62)	−2.29 (81.85, 79.56)	0.0 (+1.4, +1.4)	−0.4 (+0.6, +0.2)
P8	+1.34	+1.8	+1.1	−0.15 (1.27, 1.12)	+6.3 (138.7, 145.0)	+0.72 (+1.78, +2.50)	+6.99 (88.81, 95.80)	+0.8 (0.0, +0.8)	−0.1 (+0.1, 0.0)
P9	+1.64	+2.8	+1.3	−0.12 (1.23, 1.12)	+8.3 (138.8, 147.1)	−0.39 (+2.28, +1.89)	+0.34 (83.70, 84.04)	+1.4 (−0.7, +0.7)	−0.2 (−0.2, −0.4)
P10	+1.04	+3.0	+2.9	+0.11 (0.91, 1.02)	+3.9 (142.4, 146.3)	−2.67 (+4.20, +1.53)	+28.88 (36.17, 65.05)	+0.4 (+0.3, +0.7)	−0.2 (−0.2, −0.4)
Mean (SD) *P* value^†^	+1.31 (0.35) *P* < .001	+1.6 (1.1) *P* = .004	+2.0 (1.5) *P* = .004	−0.07 (0.14) *P* = .173	+6.5 (1.9) *P* < .001	−0.21 (1.59) *P* = .681	+1.58 (11.96) *P* *=* .686	+0.4 (0.73) *P* = .188	−0.2 (0.21) *P* = .074
Median *P* value^††^	+1.21 *P* = .002	+1.9 *P* = .012	+1.9 *P* = .008	−0.10 *P* = .205	+6.8 *P* = .002	−0.06 *P* = .770	+0.77 *P* = .695	+0.4 *P* = .125	−0.1 *P* = .109

There was a statistically significant increase in HtSDS, HV, and PAH over the 3 years of treatment with rhGH, but no statistically significant increase in bone age advancement, worsening of disproportionality, or change in BMI or bone density.

Abbreviations: BMD HAZ, bone mineral density height-adjusted *z*-score; BMI, body mass index; HtSDS, height SD score; LS, lumbar spine; N/A, not available; SH, sitting height; TBLH, total body less head.

^*a*^Δ (baseline, third year).

^†^Paired *t*-test.

^††^Signed-rank test.

Bone age advancement overall did not significantly change, as shown in Table 2, within the context of 6 of 10 participants remaining prepubertal throughout the study. Median ΔBA/CA was −0.10, without statistically significant change (*P* = .205). However, for the 4 patients who entered puberty, we started to see an increase in rate of skeletal maturation with ΔBA/CA between years 2 and 3 ranging from 0 and +0.14 compared to −0.11 and −0.01 between year 1 and year 2. Two of 10 subjects demonstrated a rate of BA maturation that outpaced CA over 3 years (patient [P] 4 and P10). This occurred in P4 in the setting of excessive weight gain in an individual with baseline obesity (BMI percentile increased from 98.79 at baseline to 99.99 after 3 years), and P10 reaching menses. Four of the eldest participants, all female, entered puberty during the study: P7 at ±8.8 years (early Tanner III breast development at end of study, no menarche yet before completion of the trial at 9.9 years), P8 at ±9.9 years (Tanner III breast development at end of study, no menarche yet before completion of the trial at 10.5 years), P9 at ±9.3 years (Tanner IV breast development at end of study, menarche at ±11 years), and P10 at ±10.5 years (Tanner IV breast development at end of study, menarche at ±12.6 years). P9 demonstrated increased rate of BA maturation between years 2 and 3 compared to between years 1 and 2 (ΔBA of 1.5 years vs 6 months, respectively), and continued with an increased pace of BA maturation postmenarche after trial completion (BA advancement of 1 year over 6-month duration by local assessment), corresponding to an updated PAH SDS of −3.4. P10 similarly showed increased pace of BA maturation postmenarche upon local assessment posttrial, with a BA of 15 years reported at CA 13.9 years, corresponding with an updated PAH SDS of −3.1.

Although all patients were started on an rhGH dose of 50 mcg/kg/day subcutaneously, 8 subjects underwent a protocol-driven dose reduction based on elevated IGF-I concentrations. After 1 year of treatment, the mean dose was 41 mcg/kg/day, 35 mcg/kg/day after 2 years, and 32 mcg/kg/day after 3 years. The median pretreatment IGF-I SDS was +0.3 (range, −0.7 to +1.1). This subsequently increased to +2.3 (range, +1.1 to +4.4) after 1 year of treatment with rhGH, +2.5 (range, +1.2 to +5.2) after 2 years, and +2.1 (range, +1.2 to +2.7) after 3 years (*P* = .002). The median IGFBP-3 SDS was −0.1 (range, −1.4 to +1.0) at baseline and increased to +0.9 (range, −0.5 to +2.3) at 3 years.

### Joint Findings and Bone Density

At baseline, 3 patients had reported intermittent joint discomfort (hip discomfort in P7, knee discomfort in P1, stiff and painful knees in P9) or weakness on examination (hip weakness in P8). There was no worsening of these complaints while on rhGH therapy, other than occasional new reports of knee and hip discomfort in P8 and occasional ankle discomfort in P7 in the first year, with decreased complaints in the latter part of the study (and no concerning examination features). One subject (P9) demonstrated clinically silent unilateral osteochondritis dissecans (OD) on magnetic resonance imaging during the study, whereas P10 was incidentally found to have OD on local imaging after suffering a possible anterior crucial ligament tear with a sports injury. P7 was found to have OD after completion of the study. Seven patients had bone densitometry by dual x-ray absorptiometry completed at baseline, which were all normal, and no significant change over treatment course was observed, with median change in lumbar spine height-adjusted *z*-score of +0.4 (*P* = .125) and total body less head height-adjusted *z*-score of −0.1 (*P* = .109) (Table 2).

### Adverse Events

There were no adverse events related to rhGH therapy, including no concerns for increased intracranial pressure, slipped capital femoral epiphysis, scoliosis, or noticeable clinical worsening of joint problems.

## Discussion

Our present study prospectively describes 3-year rhGH monotherapy in a cohort of ACAN-deficient individuals, which extends observations previously reported after 1 year of therapy [12]. Treatment with rhGH was well-tolerated throughout all 3 years of therapy without significant adverse effects or tangible change in joint manifestations. Three patients were diagnosed with OD (2 during the trial and 1 after completion of the trial), a known potential phenotypic manifestation with ACAN deficiency [6, 9, 28]. No other joint concerns were appreciated and there was no consistent change in disproportionality or bone density. There appeared to be a larger impact on HV in younger patients. The largest catch-up growth was in the first year of GH therapy, less in the second and third years, although HVs remained above baseline and normal for age. A more robust response in the first year of treatment, followed by more modest growth in the ensuing years has similarly been seen in studies describing patients with idiopathic short stature [29, 30]. Over the 3 years, there was an increase in PAH and no worsening of advanced skeletal maturation, in the context of only 4 of 10 patients entering puberty before the end of the study.

Select prior studies from the literature are briefly summarized in Table 3 for comparison. This study demonstrated a larger growth impact in the first year of treatment than observed in retrospective studies in which different rhGH doses were used [6, 19]. Deng et al describe a cohort of 5 patients treated with rhGH who demonstrated an increase in HtSDS from −3.28 ± 0.51 to −2.82 ± 0.34 with variable rhGH dosing (they also excluded 1 male patient who started during puberty at 14.7 years, without change in HtSDS after 0.4 years treatment) [10]. Retrospective data from Gkourogianni et al included patients receiving 1 of 3 different rhGH doses, with some also receiving a GnRH agonist. Mean ΔHtSDS was +0.4 after 1 year, compared to +0.74 in our cohort. Three patients in a case series reported by Xu et al each received a different dose of rhGH, with 2 subjects also treated with a GnRH agonist. The ΔHtSDS reported during the first year ranged from +0.23 to +0.33 [19]. Similar observations included a decreased benefit after the first year of rhGH therapy, despite the normal onset of puberty in some patients and the use of puberty blockade in others [6, 7, 16, 19]. The median ΔHtSDS thus far reported by Wu et al (+0.6; range, −0.1 to +1.1) was more comparable to our study; however, with only 3 of the 7 in the cohort treated for at least 1 year [18]. Sun et al reported a better response to rhGH in a cohort of 7 patients followed prospectively after identification of heterozygous *ACAN* status, with an increase in HtSDS from −2.89 ± 0.68 to −1.91 ± 0.93 [20]. However, the treatment duration was only 1.85 ± 1.91 years in a younger cohort (age range at start of 2.9-9.4 years), with only 1 patient entering puberty during follow-up.

**Table 3.** Summary of select studies describing the linear growth response in patients with aggrecan deficiency treated with recombinant human GH (rhGH), with or without addition of another growth targeted intervention (ie, GnRH and/or aromatase inhibitor)

Study	Number of patients	Median age at start (year)	rhGH Dosing range	Duration of rhGH therapy (year)	GnRHa and/or AI treatment (number treated)	Linear growth response
Muthuvel et al	10	5.6 (range, 2.4-9.7)	Initial: 50 mcg/kg/day End of year 1: 41 mcg/kg/day End of year 2: 35 mcg/kg/day End of year 3: 32 mcg/kg/day	3	None	Median baseline HtSDS: −2.52 (range, −4.27 to −1.07) Median ΔHtSDS Year 1: +0.62 (range, +0.35 to +1.39) Year 2: +0.39 (range, +0.21 to +0.55) Year 3: +0.13 (range, −0.11 to +0.53) Cumulative: +1.21 (range +0.82 to +1.94)
Deng et al (2022) [10]	5 (excluding a 14.7 year patient)	7.4 (range, 6-11.8)	33-62.7 mcg/kg/day	0.3-1.1	None	Mean baseline HtSDS: −3.28 ± 0.51 Mean HtSDS at last follow-up: −2.82 ± 0.34
Gkourogianni et al (2017) [6]	14	±7.9 (range, ±3.2 to 12.0)	30-50 mcg/kg/day	±0.5-3	GnRHa: 5 AI: 1	Mean cumulative ΔHtSDS In year 1 (14 patients): +0.4 After year 2 (4 patients): +0.7 After year 3 (2 patients): +1.0
Mancioppi et al (2021) [21]	2	9.6 (range, 8.2-11)	±35.7-38.6 mcg/kg/day	≥3	GnRHa: 2	ΔHtSDS in younger patient: +0.6 Ht in older patient at 14.7 years: 143 cm, compared to affected mother at AH of 147.3 cm
Renes et al (2023) [31]	36	9.7 (range, 3.3-14.9)	Majority ±46 mcg/kg/day (range, 33-67 mcg/kg/day)	1-3	GnRHa: 8 AI: 3 (2 treated with combined therapy)	Prepubertal patients (22) Median baseline HtSDS: −2.6 (IQR −3.5 to −2.3) Median ΔHtSDS after 1 year: +0.6 (IQR +0.5 to +0.8) Median cumulative ΔHtSDS after 3 years (10 patients): +1.0 (IQR +0.9 to +1.4) Pubertal patients (14) Median baseline HtSDS: −2.6 (IQR −3.1 to −2.0) Median ΔHtSDS after 1 year +0.3 (IQR +0.2 to +0.3) Median cumulative ΔHtSDS after 3 years (7 patients): +0.5 (IQR +0.2 to +0.7) 10 patients reached AH: 4 reached a HtSDS ≥ −2
Sun et al (2022) [20]	7	3.4 (range, 2.9-9.4)	43.3-60 mcg/kg/day	0.5-5.25	None	Mean baseline HtSDS: −2.89 ± 0.68 Mean HtSDS at last follow-up: −1.91 ± 0.93
Wu et al (2022) [18]	7	7.0 (range, 2.9-14.3)	40-57 mcg/kg/day	0.1-1.5	AI: 1	Median baseline HtSDS: −2.1 (range, −4.9 to −0.7) Median ΔHtSDS: +0.6 (range −0.1 to +1.1)
van der Steen et al (2017) [7]	4	11.8 (range, 5.0-12.3)	1-2 mg/m^2^/day	±3.5-9	GnRHa: 4 AI: 2 (after discontinuation of GnRHa)	Median baseline HtSDS: −2.7 (range, −3.7 to −2.4) ΔHtSDS of +0.7 in 1 patient (±3.5 years of rhGH) ΔHtSDS ranged from −0.2 to +0.1 in remaining 3 patients 2 patients reached AH: HtSDS of −3.9 and −2.6, taller than their affected parents (ΔHtSDS +0.8 and +1.2, respectively)
Xu et al (2018) [19]	3	6.4 (range, 5.6-7.8)	40-60 mcg/kg/day	3.9-8	GnRHa: 2	Median baseline HtSDS: −2.09 (range, −3.74 to −0.88) Median ΔHtSDS Year 1: +0.27 (range, +0.23 to +0.33) Cumulative: +0.57 (range, −0.01 to +1.02)

Abbreviations: AH, adult height; AI, aromatase inhibitor; HtSDS, height SD score; IQR, interquartile range; rhGH, recombinant human GH.

Three-year results in this study for prepubertal patients were similar to recently published work by Renes et al describing a cohort of 36 patients (22 prepubertal) with ACAN deficiency in the Dutch National Registry of GH Treatment who were treated with an rhGH dose of ± 46 mcg/kg/day (8 patients born SGA were already on 33 mcg/kg/day, 2 patients already treated per a study protocol for SGA with 67 mcg/kg/day, 1 patient with increased dosing because of suboptimal response, and 5 patients with dose reductions from elevated IGF-I) [31]. As in Table 3, of the 22 prepubertal subjects, all were treated with rhGH for at least 1 year, and 10 patients for 3 years, with median cumulative ΔHtSDS of +0.6 and +1.0, respectively [31], comparable to the median ΔHtSDS of +0.62 in the first year of our study, and +1.21 after 3 years. Although lower rhGH dosing was used in our study because of IGF-I-driven dose reductions, growth response was similar.

Renes et al also included 14 pubertal subjects, 7 of whom were treated for 3 years with rhGH (additionally with GnRH agonist therapy and/or an aromatase inhibitor), with median ΔHtSDS of +0.5 [31]. However, of the 10 patients in the total cohort who reached adult height, only 4 reached a HtSDS ≥−2, and 5 reached an adult height greater than their affected parent [31]. Related to this, van der Steen et al reported on 2 of 4 patients born small for gestational age with pathogenic ACAN variants who were treated with rhGH (dose range, 1-2 mg/m^2^/day and duration ranging from ±3.5 to 9 years), plus GnRH agonist therapy, who reached adult height, but without showing significant change in HtSDS (ΔHtSDS of −0.2 and +0.1), albeit still taller than their affected parents (HtSDS +0.8 and +1.2 compared to the parents after ±9 and 6 years of rhGH treatment, respectively) [7]. Mancioppi et al also reported on 2 sisters treated with rhGH and GnRH agonist therapy, with 1 showing ΔHtSDS of +0.6 after 3.4 years of rhGH (0.27 mg/kg/week started 8.2 years) and 1 year of puberty blockade, but her elder sister only reaching a height of 143 cm at 14.7 years (and reaching menarche) after 3.7 years of rhGH (0.25 mg/kg/week started at 11 years) and 3 years of GnRH agonist therapy (initiated at approximately 9.8 years), compared to their untreated mother at an adult height of 147 cm [21]. In this trial, 2 pubertal patients for whom near-adult height measurements were made available posttrial reached adult or near-adult HtSDS of <−3 SD and not necessarily above their untreated ACAN-deficient parent. Advanced BA did not worsen during the 3 years of treatment with rhGH (however, more than half of the participants remained prepubertal). Nevertheless, rapid BA maturation during puberty leading to earlier growth cessation may attenuate the impact on adult height outcome compared to PAH based on the initial growth response and BA assessments (the latter not necessarily reliable in the setting of a skeletal disorder). Starting at a younger age (early-mid childhood) may improve long-term height outcome because of a robust first-year response and longer duration for potential prepubertal catch-up growth (or, at minimum, more years in prepuberty with a normal HV for age). Although pubertal blockade may extend the duration during which a growth-promoting agent can be used, at this point it is unclear how much impact such has on adult height, particularly if rhGH is not started before onset of puberty or peripubertal time frame. It will be important to follow those started on rhGH at a younger age through puberty to determine the ultimate impact on adult height.

This study was limited by relatively small sample size for a thus-far underrecognized emerging genetic short stature condition. The sample size limits ability to make clear correlations between genotype and rhGH response. Furthermore, there is a phenotypic spectrum with ACAN deficiency, including magnitude of growth plate involvement and short stature, likely contributing to rhGH response heterogeneity and impacting the generalizability of results. Additionally, without follow-up through puberty and adult height, it is difficult to definitively conclude the final impact of rhGH, as well as monitor for long-term adverse effects. Metabolic markers were not monitored given similar initial rhGH dosing to other primary skeletal disorders; however, if above the mean IGF-I levels were to persist, it may be beneficial to monitor over time.

In this study, improvement in linear growth was demonstrated on modest rhGH dosing in a cohort of patients with short stature because of ACAN deficiency. The largest growth response was observed during the first year, followed by at least maintaining a normal-for-age HV and above the pretreatment HV afterwards. Caution should be exercised in estimating the impact on adult height outcome from the early treatment response, particularly in those individuals for whom rhGH is only initiated in the peripubertal period. Given the current body of evidence, therapy with rhGH in ACAN-deficient short children is effective and safe, but more adult height outcome data are needed to better qualify the overall benefit of this intervention.

## Data Availability

Some or all data sets generated during and/or analyzed during the current study are not publicly available but are available from the corresponding author on reasonable request.

## Clinical Trial Information

This study is registered on ClinicalTrials.gov (NCT03288103).

## Abbreviations

ACAN: aggrecan
BA: bone age
BMI: body mass index
CA: chronologic age
HtSDS: height SD score
HV: height velocity
IGFBP-3: IGF binding protein-3
OD: osteochondritis dissecans
P: patient
PAH: predicted adult height
rhGH: recombinant human GH
SDS: SD score
SH/Ht: sitting height/standing height
